# Secretome of atrial epicardial adipose tissue facilitates reentrant arrhythmias by myocardial remodeling

**DOI:** 10.1016/j.hrthm.2022.05.011

**Published:** 2022-05-12

**Authors:** Auriane C. Ernault, Arie O. Verkerk, Jason D. Bayer, Kedar Aras, Pablo Montañés-Agudo, Rajiv A. Mohan, Marieke Veldkamp, Mathilde R. Rivaud, Rosan de Winter, Makiri Kawasaki, Shirley C.M. van Amersfoorth, Eva R. Meulendijks, Antoine H.G. Driessen, Igor R. Efimov, Joris R. de Groot, Ruben Coronel

**Affiliations:** * Department of Clinical, Experimental Cardiology and Medical Biology, Amsterdam UMC, Amsterdam, The Netherlands,; † IHU-LIRYC, Electrophysiology and Heart Modeling Institute, Bordeaux University Foundation, Pessac, France,; ‡ Centre National De La Recherche Scientifique, Institut de Mathématiques de Bordeaux, UMR5251, Bordeaux, France,; § Department of Biomedical Engineering, George Washington University, Washington, DC,; ¶ Department of Biomedical Engineering, Northwestern University, Chicago, Illinois.

**Keywords:** Atrial fibrillation, Arrhythmias, Epicardial adipose tissue, Ion channels, Obesity

## Abstract

**BACKGROUND:**

Epicardial adipose tissue (EAT) accumulation is associated with cardiac arrhythmias. The effect of EAT secretome (EATs) on cardiac electrophysiology remains largely unknown.

**OBJECTIVE:**

The purpose of this study was to investigate the arrhythmogenicity of EATs and its underlying molecular and electrophysiological mechanisms.

**METHODS:**

We collected atrial EAT and subcutaneous adipose tissue (SAT) from 30 patients with atrial fibrillation (AF), and EAT from 3 donors without AF. The secretome was collected after a 24-hour incubation of the adipose tissue explants. We cultured neonatal rat ventricular myocytes (NRVMs) with EATs, subcutaneous adipose tissue secretome (SATs), and cardiomyocytes conditioned medium (CCM) for 72 hours. We implemented the electrophysiological changes observed after EATs incubation into a model of human left atrium and tested arrhythmia inducibility.

**RESULTS:**

Incubation of NRVMs with EATs decreased expression of the potassium channel subunit *Kcnj2* by 26% and correspondingly reduced the inward rectifier K^+^ current I_K1_ by 35% compared to incubation with CCM, resulting in a depolarized resting membrane of cardiomyocytes. EATs decreased expression of connexin43 (29% mRNA, 46% protein) in comparison to CCM. Cells incubated with SATs showed no significant differences in *Kcnj2* or *Gja1* expression in comparison to CCM, and their resting potential was not depolarized. Cardiomyocytes incubated with EATs showed reduced conduction velocity and increased conduction heterogeneity compared to SATs and CCM. Computer modeling of human left atrium revealed that the electrophysiological changes induced by EATs promote sustained reentrant arrhythmias if EAT partially covers the myocardium.

**CONCLUSION:**

EAT slows conduction, depolarizes the resting potential, alters electrical cell–cell coupling, and facilitates reentrant arrhythmias.

## Graphical Abstract

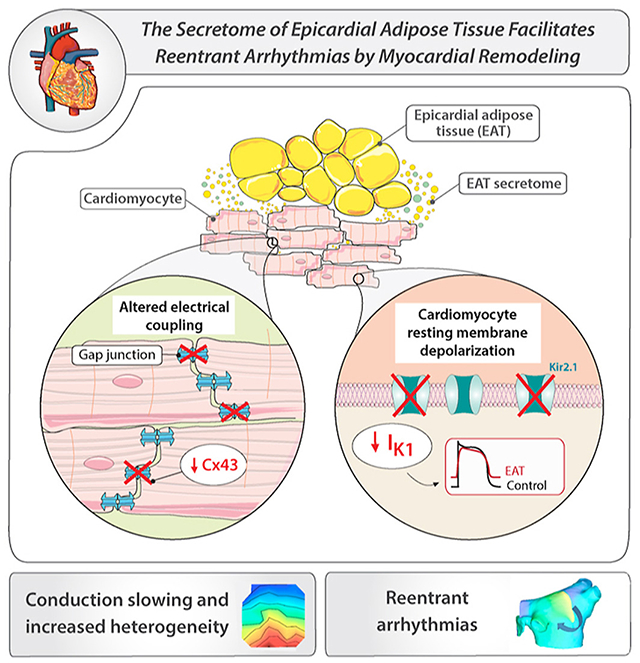

## Introduction

Obesity and overweight are substantial and growing public health challenges resulting in more than 1 million deaths per year in Europe.^1^ Obesity is an independent risk factor for sudden cardiac death and atrial fibrillation (AF).^2^ In obese individuals, adipose tissue (AT) accumulates around internal organs such as the heart. Greater volumes of epicardial adipose tissue (EAT) are present in patients with persistent AF than in those with paroxysmal AF,^3^ suggesting that EAT facilitates the maintenance of sustained arrhythmias.

In view of the proposed relationship between EAT and arrhythmogenesis, several studies investigated the mechanisms underlying the arrhythmogenicity of EAT.^4^ EAT secretes proinflammatory adipokines and extracellular vesicles, which participate in creating a substrate for arrhythmias.^5^ Moreover, epicardial adipose tissue secretome (EATs) induces direct ionic remodeling: EATs isolated from rabbit hearts induces acute changes in ion currents in rabbit atrial cardiomyocytes, leading to a longer action potential (AP) duration (APD) at 90% repolarization (APD_90_) and a depolarized resting membrane (RM).^6^ Also, sheep EAT fragments prolong the field potential duration (surrogate measure for APD) of human induced pluripotent stem cell–derived cardiomyocytes (hiPSC-CM).^7^ However, the consequences of such ionic modifications on cardiac conduction and arrhythmogenesis are unknown. Mouse pericardial (ie, thoracic) AT-derived secretome exerts little effect on conduction in HL-1 cells,^7^ but these cells do not present a physiological conduction velocity (CV). Moreover, EAT likely exerts different electrophysiological consequences than pericardial AT because of distinct biomolecular features.^8^

The aim of this research was to examine the effects of prolonged exposure (72 hours) to human EATs on cardiac conduction in a cell culture model with normal CV and on arrhythmogenesis in an *in silico* model of human left atrium (LA). We hypothesized that EATs induces electrical remodeling of the myocardium, facilitating arrhythmias. Therefore, we compared the effect of human EATs with subcutaneous adipose tissue (SAT) secretome (SATs) on the electrophysiological characteristics of cultured neonatal rat ventricular myocytes (NRVMs). We also studied changes in ion channel expressions underlying the observed electrical remodeling. Finally, we implemented the electrophysiological changes induced by EATs into a model of human LA and tested arrhythmia inducibility.

## Methods

A detailed version of the Methods is available in the Supplemental Material.

### Human AT collection

Thirty patients with symptomatic, persistent, or long-standing persistent AF were recruited at the Amsterdam UMC, University of Amsterdam. EAT was harvested from the LA appendage upon excision. Written informed consent was obtained from all participants. The study was conducted in accordance with the Declaration of Helsinki as revised in 2013 and with the Medical Research Involving Human Subjects Act (WMO), and was approved by the institutional review board of the Amsterdam AMC (for details, see Supplemental Material). The clinical characteristics of patients are listed in Supplemental Table 1.

### Statistical analysis

Statistical analyses were performed using GraphPad Prism (GraphPad Software, San Diego, CA). Data are given as mean ± SD unless indicated otherwise and were analyzed with appropriate statistical tests, as indicated in the respective figure legends. Number of observations and repeated experiments are given in the figure legends. Data were tested for normality using a Shapiro-Wilk normality test. If normal distribution was valid, the 2-tailed Student t test was performed. If the data were not normally distributed, statistical significance was measured using the 2-tailed Mann-Whitney test. For multiple comparisons, a Kruskal-Wallis test was performed, followed by the Dunn post-test. *P* ≤.05 was considered significant.

## Results

### EAT slows conduction and depolarizes the RM of cardiomyocytes

**Figure 1:**
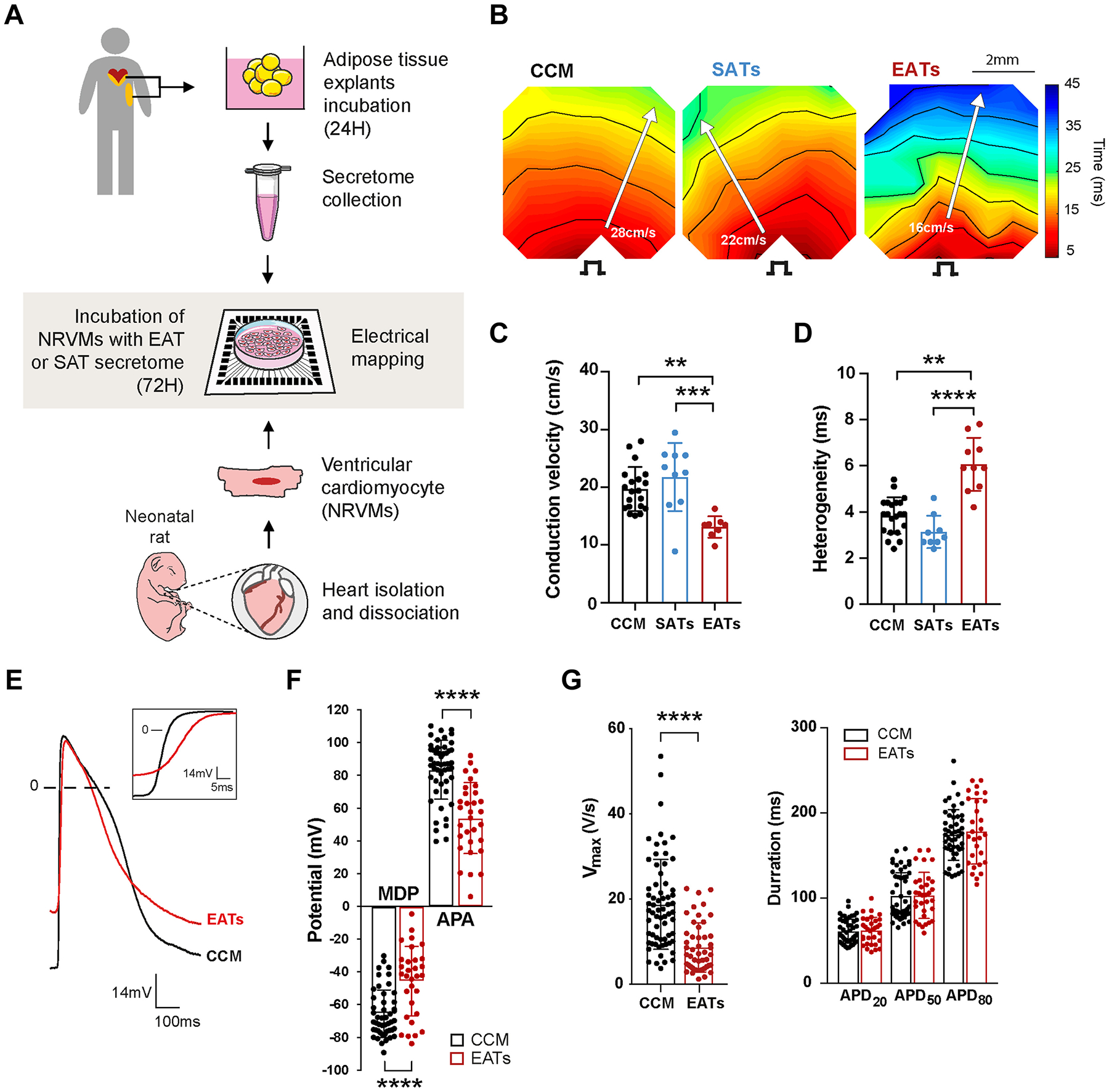
Epicardial adipose tissue secretome (EATs) slows conduction, increases heterogeneity, and depolarizes cardiomyocytes. **A:** Experimental outline of the electrophysiological study. Epicardial adipose tissue (EAT) and subcutaneous adipose tissue (SAT) were harvested, dissected, and incubated as explants in culture medium for 24 hours before collection of its secretome. Neonatal rat ventricular myocytes (NRVMs) were seeded to form monolayers on multielectrode arrays and incubated for 72 hours with the adipose tissue secretome before electrical mapping. A summary of the EATs and subcutaneous adipose tissue secretome (SATs) samples used for each subpart of the study can be found in Supplemental Table 2. **B:** Representative activation maps obtained after electrical mapping of NRVMs incubated for 72 hours with cardiomyocytes conditioned medium (CCM), SATs, or EATs. Colors indicate activation relative to the stimulus artifact times, according to the scale at right. Isochrones = 5 ms. Stimulus symbol indicates stimulation site. **C, D:** Conduction velocity (**C**) and heterogeneity (**D**) in paced cardiac monolayers after incubation with CCM, SATs, or EATs. Data are given as mean ± SD. n ≥8 monolayers from 4 independent NRVMs isolation. Kruskal-Wallis test followed by the Dunn post-test. **E:** Representative spontaneous action potentials (APs) recorded in cardiac monolayers incubated with EATs or CCM. Maximum diastolic potential (MDP) is less negative in EATs than in CCM. **Inset** shows the reduction of AP upstroke after EATs. **F, G:** Effect of EATs incubation on spontaneous APs, MDP, action potential amplitude (APA) (**F**), maximal upstroke velocity (V_max_), and action potential duration at 20%, 50%, and 80% (APD_20_, APD_50_, and APD_80_) of repolarization (n ≥29 microelectrode measurements from 4 independent NRVMs isolation) (**G**). Data are given as mean 6 ± SD. Nonparametric Mann-Whitney test. **P <.01; ***P <.001; ****P <.0001.

To study the effect of AT secretome on the electrophysiological properties of cardiomyocytes, we performed electrical mapping on NRVMs incubated with EATs, SATs, and cardiomyocyte conditioned medium (CCM) for 72 hours (Figure 1A). Activation maps obtained from NRVMs exposed to EATs revealed heterogeneous isochrone crowding, indicating slower conduction in EATs but not in SATs or CCM (Figure 1B). Overall, CV was 19.7 ± 3.8 cm/s and 21.8 ± 5.9 cm/s after exposure to CCM and SATs, respectively, but it was 13.1 ± 1.8 cm/s after exposure to EATs (Figure 1C). EATs significantly increased conduction heterogeneity in comparison to SATs and CCM (Figure 1D). Conduction heterogeneity was 3.8 ± 0.8 ms, 3.1 ± 0.7 ms, and 6.1 ± 1.1 ms after exposure to CCM, SATs, and EATs, respectively. No significant difference was observed between CCM and SATs.

To understand whether the observed conduction slowing was linked to a pro-apoptotic effect of EATs and a loss of confluence of the NRVMs monolayers, we performed live-cell analysis and measured cell viability in NRVMs incubated for 72 hours with EATs. Annexin V was used as a probe to measure phosphatidylserine exposure during apoptosis. YOYO-3, a membrane-impermeable DNA dye, was used to measure permeabilized apoptotic and dead cells. Cell confluence was quantified at 0 and 72 hours by phase contrast. We found that acute (0 hour) and prolonged (72 hours) exposure to EATs did not alter cell confluence (Supplemental Figure 1A). Apoptosis was significantly decreased in the cells incubated for 72 hours with EATs in comparison to CCM (Supplemental Figure 1B), whereas there were no significant differences in cell death between the 2 groups (Supplemental Figure 1C).

Because EAT evolves in the context of a disease,^9^ we next investigated whether the observed CV slowing induced by EATs in AF patients depends on the presence of AF. We collected EATs from the hearts of 3 donors without AF and performed the same experiments as shown in Figures 1A to 1D. Activation maps obtained by electrical mapping of NRVMs revealed that EATs from non-AF hearts induces isochrone crowding and CV slowing (Supplemental Figure 2A). Overall, EATs from non-AF hearts decreased conduction (22.9 ± 3.2 cm/s control vs 14.8 ± 3.3 cm/s EATs non-AF) (Supplemental Figure 2B) and increased heterogeneity (3.3 ± 0.4 ms control vs 5.0 ± 0.95 ms EATs non-AF) (Supplemental Figure 2C).

Representative APs from NRVMs monolayers exposed to CCM and EATs are shown in Figure 1E. NRVM exposed to EATs presented a less negative maximum diastolic potential (MDP), reduced action potential amplitude (APA), and reduced maximum upstroke velocity (V_max_) compared to CCM (Figure 1E). The averaged MDP after exposure to EATs was 19.9 mV less negative than CCM (−65.5 ± 14.5 mV CCM vs –45.6 ± 21.2 mV EAT) (Figure 1F). This depolarization was accompanied by a 29.4-mV decrease in APA (83.4 ± 17.9 mV CCM vs 54.0 ± 21.7 mV EAT) (Figure 1F) and a 10.1-V/s reduction of V_max_ (18.7 ± 10.6 V/s CCM vs 8.6 ± 5.8 V/s EAT) (Figure 1G). There were no significant differences in APD (Figure 1G) or spontaneous cycle length (not shown) between EATs and CCM.

### EATs-induced depolarization due to reduction of inward rectifier K^+^ current

**Figure 2:**
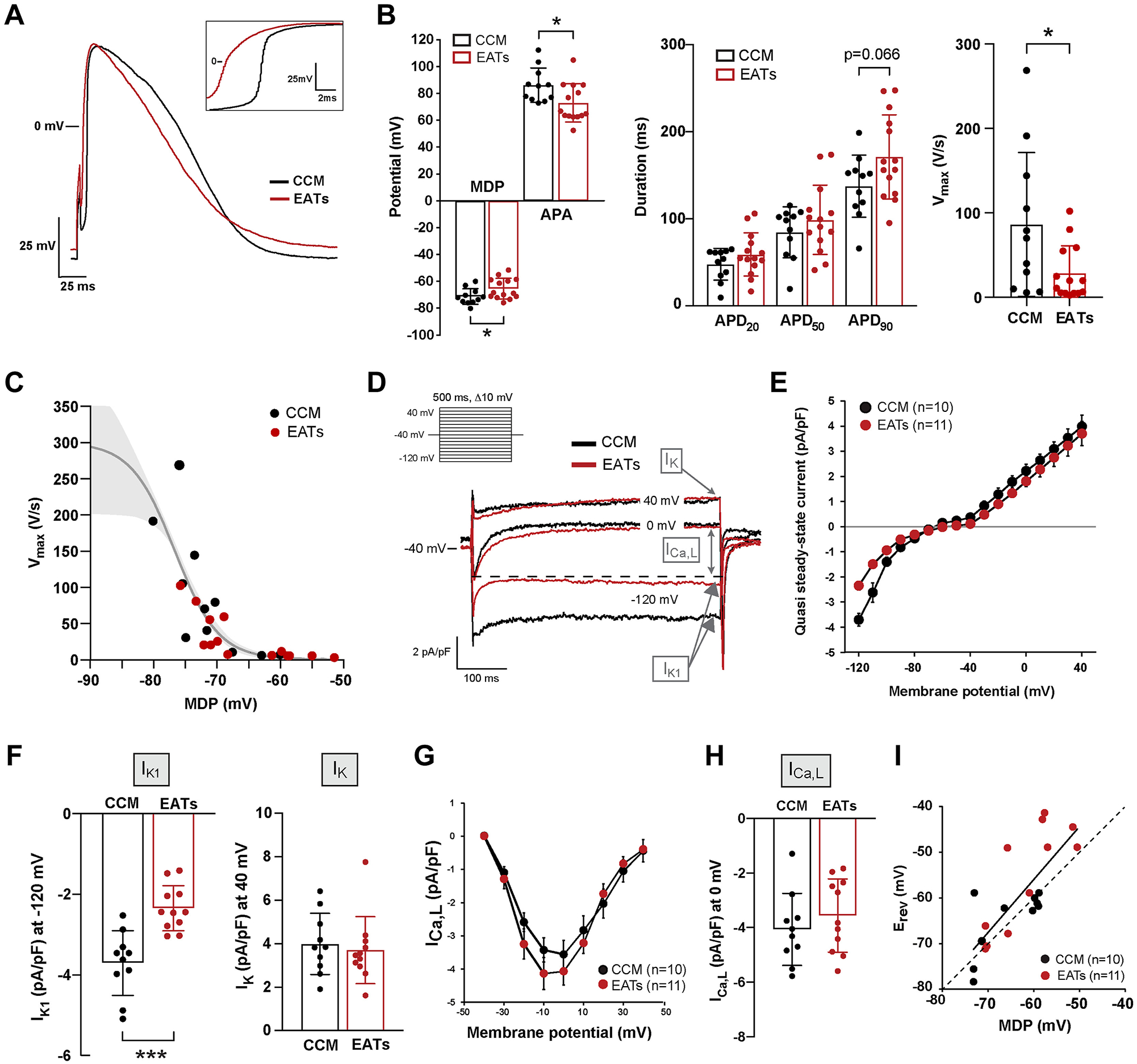
Epicardial adipose tissue secretome (EATs) alters the electrophysiological characteristics of single cardiomyocytes by reducing the inward rectifier K^+^ current I_K1_. **A:** Representative elicited action potentials (APs) (4 Hz) recorded in single cardiomyocytes. **Inset** shows the reduction of the AP upstroke after EATs. **B:** Average characteristics of APs elicited at 4 Hz: maximal maximum diastolic potential (MDP) and action potential amplitude (APA), action potential duration at 20%, 50%, and 90% (APD_20_, APD_50_, APD_90_), and maximal upstroke velocity (V_max_). Data are given as mean ± SD. n ≥13 cells from 4 independent neonatal rat ventricular myocytes (NRVMs) isolation. Student *t* test for MDP, APA, APD_90_; nonparametric Mann-Whitney for V_max_. **C:** V_max_-MDP relationship where the *gray solid line* indicates the Boltzmann function y = 300/[1 +*exp*{(*V* − *V*_1/2_)/*k*}], indicating that the reduced V_max_ in EATs cells is due at least in part to reduced Na^+^ channels availability caused by the depolarization. **D:** Representative currents tracing measured at −120, 0, and +40 mV. **Inset:** voltage protocol. *Dashed line* indicates the peak of the L-type Ca^2+^ current I_Ca,L_. **E:** Average current–voltage (I–V) relationships of the quasi steady-state current measured at the end of the voltage-clamp steps. Data are given as mean ± SEM. **F:** Current density of the quasi steady-state current measured at −120 mV (defined as I_K1_) and at 40mV (defined as I_K_). Data are given as mean ± SD. n ≥10 cells from 4 independent NRVMs isolation. Student *t* test. **G:** Average I–V relationship of I_Ca,L_. Data are given as mean ± SEM. **H: I**_Ca,L_ density at 0 mV, defined as I_Ca,L_. Data are given as mean ± SD. n ≥10 cells from 4 independent NRVMs isolation. **I:** MDP-E_rev_ relationship indicating the strong relation between these parameters. *Dashed line* indicates the line of identity. *P <.05; ***P <.001. CCM = cardiomyocytes conditioned medium.

We next performed perforated patch-clamp experiments to record paced (Figure 2) and spontaneous (Supplemental Figure 3) APs and ion currents from single NRVMs incubated for 48 hours with EATs or CCM, or with SATs or CCM (Supplemental Figure 4). To make a direct comparison between MDP and background K^+^ currents within the same cell and to ensure that AP shapes and the remaining viable cardiomyocytes in the recording chamber stay undistorted for biophysical analysis, these measurements were performed without specific channel blockers or modified solutions.

Figure 2A shows representative paced APs recorded from single cells incubated for 48 hours with EATs (red) and CCM (black), respectively. Figure 2A (inset) shows the upstroke of these APs. MDP of cardiomyocytes exposed to EATs was on average 5.9 mV less negative than that of CCM-exposed cardiomyocytes (71.3 ± 5.9 mV CCM vs −65.4 ± 7.6 mV EATs) (Figure 2B). In addition, APA was reduced by 13.2 mV in cardiomyocytes incubated with EATs (86.1 ± 12.8 mV CCM vs 72.9 ± 14.3 mV EATs), whereas there were no statistically significant differences in APD at 20% repolarization (APD_20_) and 50% repolarization (APD_50_) between groups (Figure 2B). In contrast, APD_90_ was increased after incubation with EATs (137.4 ± 35.7 ms CCM vs 171.0 ± 48.3 ms EATs; *P* = .066) (Figure 2B). V_max_ of paced APs was significantly reduced after EATs incubation (86.3 ± 85 V/s CCM vs 28.6 ± 32.3 V/s EATs) (Figure 2B) and showed a clear sigmoidal relationship with MDP (Figure 2C) as expected from the voltage-dependence of inactivation of sodium channels. Spontaneous APs showed similar changes upon exposure to EATs, whereas cycle length was not significantly different between CCM and EATs (Supplemental Figure 3). Cardiomyocytes exposed to SATs did not show significant differences in AP characteristics in comparison to CCM (Supplemental Figure 4).

Next, we performed voltage-clamp experiments on single cardiomyocytes. We applied a general voltage protocol to record inward rectifier K^+^ current (I_K1_), delayed rectifier K^+^ current (I_K_), and L-type Ca^2+^ current (I_Ca,L_) (Figure 2D, inset). Typical current recordings from CCM- and EATs-treated cardiomyocytes are shown in Figure 2D and Supplemental Figure 4. Example current tracings and average current–voltage (I–V) relationships indicate a difference in I_K1_, but not in I_Ca,L_ or in I_K_, between EATs- and CCM-treated cardiomyocytes (Figures 2D and 2E). Overall, EATs-treated cardiomyocytes presented with a 35% reduction in I_K1_ (at −120 mV) in comparison to CCM-treated cardiomyocytes (−3.7 ± 0.8 pA/pF CCM vs −2.3 ± 0.6 pA/pF EATs) (Figure 2F, left). Average I_K_ (at 40 mV) and I_Ca,L_ (at 0 mV) densities did not differ significantly between CCM and EATs (Figures 2F, right, 2G, and 2H). Average I_K1_, I_Ca_,_L_, and I_K_ densities did not differ significantly between SATs and CCM (Supplemental Figure 4). MDP and reversal potential (E_rev_) of the background K^+^ current showed a linear relationship along the line of identity (dashed line Figure 2I), which shows that the depolarization of cardiomyocytes is importantly caused by the reduction in I_K1_.

### EATs exposure leads to electrical remodeling of cardiomyocytes

**Figure 3:**
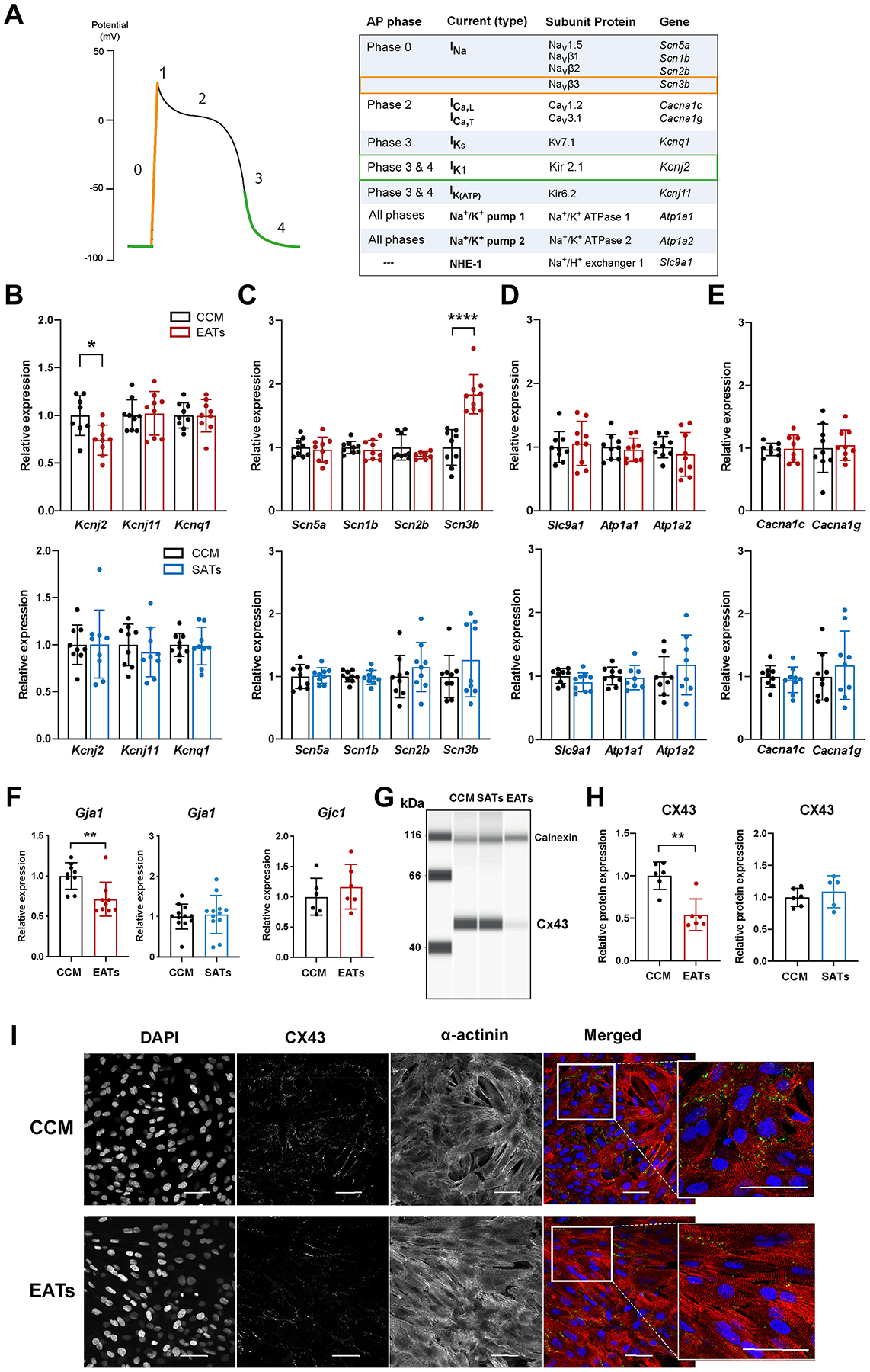
Epicardial adipose tissue secretome (EATs) induces electrical remodeling of cardiomyocytes. **A:** Schematic ventricular action potential (AP) phases and relative current contribution, subunit protein, and genes. **B–E:** mRNA levels expression of potassium (**B**), sodium (**C**), pumps and exchangers (**D**), and calcium (**E**) related genes in cardiomyocytes after incubation with EAT, subcutaneous adipose tissue secretome (SATs), or cardiomyocytes conditioned medium (CCM). Data are given as mean ± SD. EATs vs CCM: n ≥7 minimum from 3 independent neonatal rat ventricular myocytes (NRVMs) isolation. SATs vs CCM: n ≥8 minimum from 3 independent NRVMs isolation. Student *t* test and nonparametric Mann-Whitney test, respectively. Primer sequences are given in Supplemental Table 3. **F, left and middle:** Relative expression of *Gja1*, gene encoding for connexin43 (CX43), in EAT, SATs, and CCM groups, measured by real-time quantitative polymerase chain reaction PCR. Data are given as mean ± SD. EATs vs CCM: n ≥9 from 3 independent NRVMs isolation. SATs vs CCM: n = 12 from 4 independent NRVMs isolation. Nonparametric Mann-Whitney test. Primer sequences are given in Supplemental Table 3. **F, right:** Relative expression of *Gjc1*, gene encoding for connexin45 (CX45). Data are given as mean ± SD. n ≥6 from 2 independent NRVMs isolation. Primer sequences are given in Supplemental Table 3. **G:** Representative image of CX43 protein quantification in NRVMs protein lysates collected after incubation with CCM, SATs, and EATs, measured by simple western assay (protein simple). Calnexin used as loading control. Antibodies references are given in Supplemental Table 4. **H:** Quantification of CX43 protein expression in NRVMs incubated with EATs (**left**), SATs (**right**), and CCM. CX43 signal was normalized to calnexin. Data are given as mean ± SD. n ≥5 from 2 independent NRVMs isolation. Nonparametric Mann-Whitney test. Antibodies references are given in Supplemental Table 4. **I**: Immunofluorescence images of NRVMs monolayers incubated with EATs or CCM, and stained with CX43 (*green*), α-actinin (*red*), and DAPI (*blue*). Antibodies references are given in Supplemental Table 4. *P, <.05; **P <,0.01; ****P <,.0001.

To determine the basis of the electrophysiological changes observed after EATs exposure, we measured the mRNA expression of ion channel–related genes that determine MDP, APD, and CV (Figure 3A) in NRVMs incubated with EATs or CCM and, in separate experiments, in NRVMs incubated with SATs or CCM.

In the EATs group, we observed a significant decreased expression of *Kcnj2* (FC = 0.74 ± 0.16) (Figure 3B, top) in comparison to CCM, whereas SATs did not significantly change *Kcnj2* expression (Figure 3B, bottom). In contrast, *Kcnq1* and *Kcnj11* expressions were not significantly different after exposure to EATs, SATs, or CCM (Figure 3B). *Scn5a*, *Scn1b,* and *Scn2b* expressions also were not significantly different between the 2 groups, whereas the subunit *Scn3b* was significantly upregulated (FC = 1.84 ± 0.31) (Figure 3C, top) with EATs in comparison to CCM. There was no significant change in *Scn3b* expression between the SATs and CCM groups (Figure 3C, bottom). Other pumps and exchanger-encoding genes (*Slc9a1, Atp1a1, Atp1a2*) as well as calcium-related genes (*Cacna1c, Cacna1g*) were not differentially expressed in the EATs, SATs, and CCM groups (Figures 3D and 3E).

Several inflammatory adipokines in EATs can downregulate the expression of gap junctional channel protein connexin43 (Cx43, *Gja1*),^4^ which plays a major role in electrical coupling, tissue homogeneity, and cardiac conduction. Exposure of cardiomyocytes to EATs led to a significant reduction in expression of *Gja1* in comparison to CCM (FC = 0.71 ± 0.21) (Figure 3F, left), whereas *Gjc1* expression was unchanged. However, no significant difference was found in *Gja1* expression between cardiomyocytes exposed to SATs and CCM (Figure 3F, right). The observed decrease in *Gja1* expression levels with EATs was accompanied by a significant decrease in Cx43 protein expression (FC = 0.54 ± 0.19) as measured by capillary electrophoresis (Figures 3G and 3H, and Supplemental Figure 5) and supported by immunostaining (Figure 3I). Finally, Cx43 protein expression levels were not significantly different between SATs and CCM groups (Figures 3G and 3H, and Supplemental Figure 5).

### EATs facilitates sustained arrhythmias in a human LA model

We next performed computer simulations with EATs-induced electrophysiological changes to a human LA model (Supplemental Figure 6) to test the hypothesis that increasing the amount of EAT leads to increased arrhythmia inducibility. Based on imaging studies, we gradually applied EAT patches on the surface of the LA model in order to cover from 0% to 100% of the surface of the epicardium. In the epicardial regions where EAT patches were applied, we modified 3 model parameters to reproduce the changes observed after EATs incubation in the NRVM model. We first decreased I_K1_ by 35%, followed by reducing the intracellular potassium concentration until the resting membrane potential (RMP) matched experimental values. We then equally reduced the longitudinal and transverse tissue conductivities until CV in the model decreased by 25% to reproduce the slower CV in the experiments from reduced cellular coupling and Cx43 expression.

**Figure 4:**
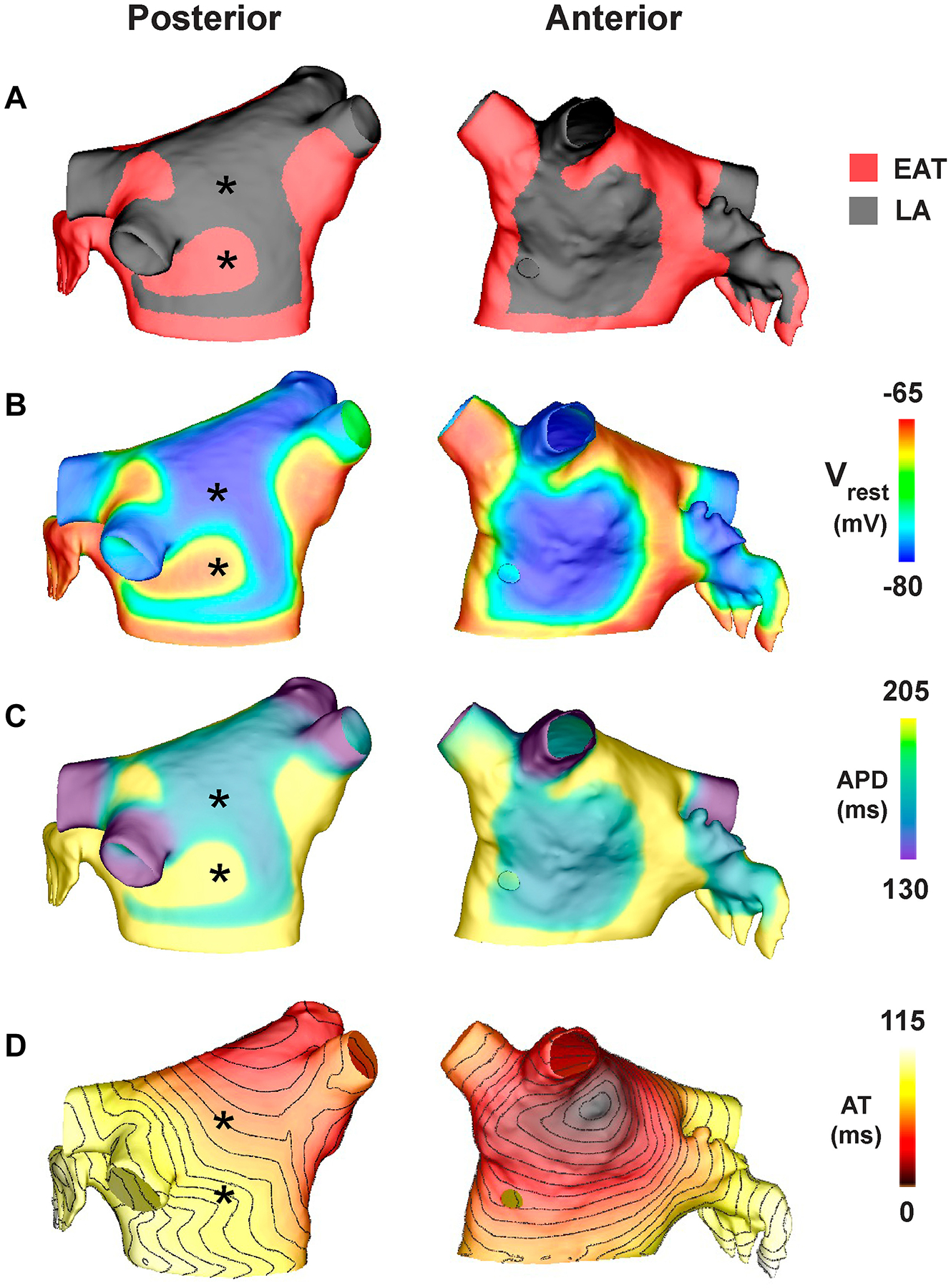
Computational model of the human left atrium (LA) electrically remodeled by epicardial adipose tissue secretome (EATs). **A:** Posterior and anterior views of the computational LA model with 50% epicardial adipose tissue (EAT) during baseline pacing, with a cycle length of 700 ms from the sinus rhythm pacing site. **B–D:** Resting membrane potential (V_rest_) (**B**), action potential duration at 80% repolarization (APD) (**C**), and activation time (AT) (**D**) with isolines every 5 ms for the last beat of pacing.

**Figure 5:**
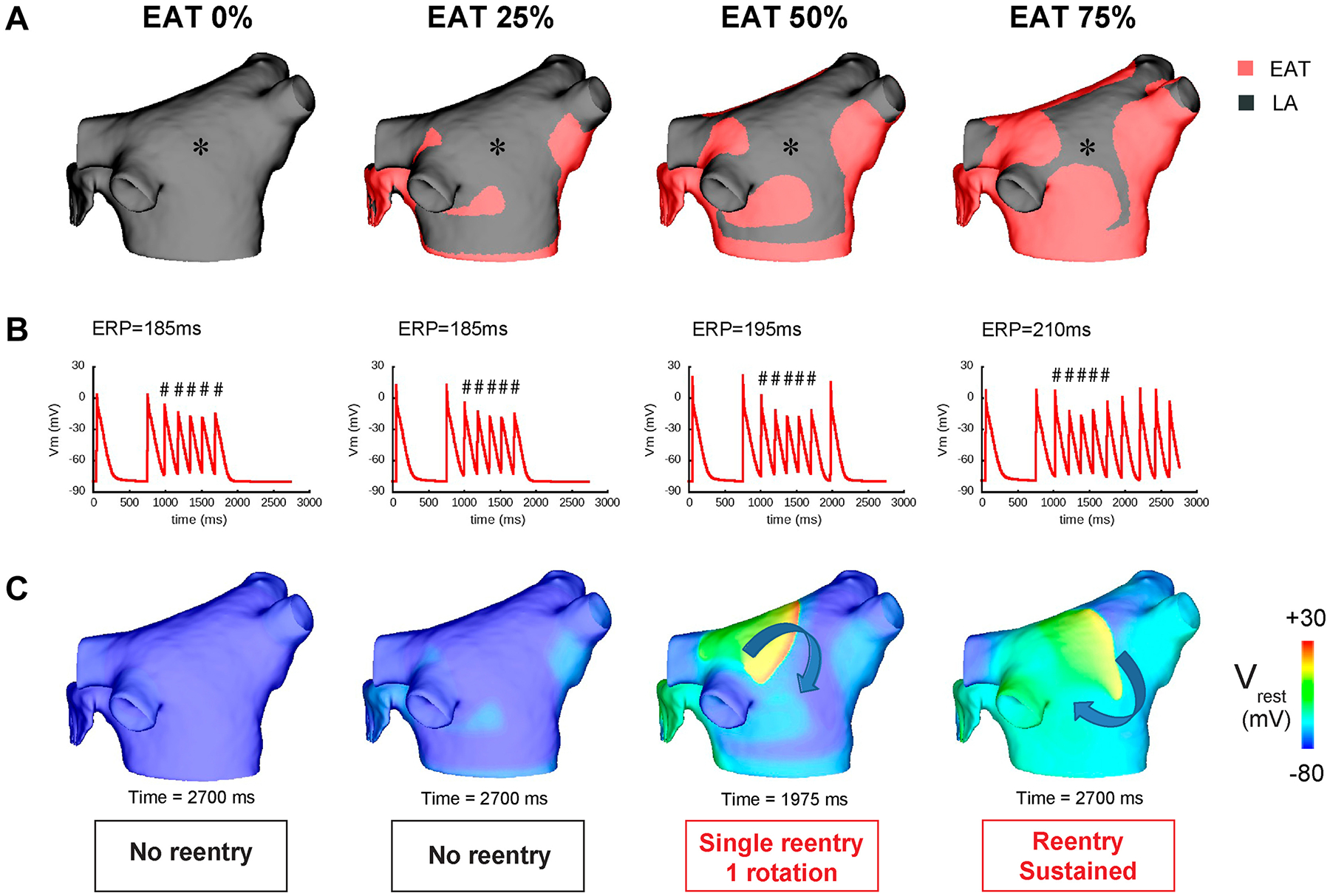
Arrhythmia vulnerability induced by epicardial adipose tissue (EAT) secretome. **A:** Posterior view of the computational left atrial (LA) model with different percentages of EAT. **B:** Membrane potential plotted at the *asterisk* indicated in **A** showing the burst pacing protocol and arrhythmia outcome. # indicates paced beat with 170-ms cycle length. **C:** Membrane potential maps at specific times following burst pacing. ERP = effective refractory period.

By decreasing I_K1_ in EAT regions (Figure 4A), we observed results similar to those observed in the cellular electrophysiological study (Figures 1 and 2). Specifically, RMP was elevated by 15.2 mV (−64.6 mV vs −79.8 mV, values at * in Figure 4B, EAT 50%), and APA decreased from 74 to 55 mV. A small increase (6%) in APD_80_ also was observed, consistent with our results in single cells (Figure 4C). The combined results showed that total LA activation time was delayed by up to 11 ms depending on the percentage of EAT on the LA (106, 107, 115, and 117 ms at EAT 0%, 25%, 50%, and 75%, respectively) (Figure 4D). Arrhythmia inducibility also depended on the percentage of EAT (Figure 5A). Reentry was observed for EAT ≥50%, with short-lived reentry occurring for 50% EAT and sustained reentry occurring for 75% EAT (Figures 5B and 5C). Sustained reentry lasted the entire duration of the simulations (10 seconds postreentry initiation). As observed in the video for Figures 5B and 5C (Supplemental Video and Supplemental Material), conduction block and reentry occurred at the interface between the EAT and non-EAT regions. We did not observe reentry with 100% EAT, suggesting that conduction heterogeneity plays an important role in EATs-induced arrhythmia vulnerability. In the control LA model without AF remodeling to shorten APD, we observed very short-lived reentry (1–2 rotations) during burst pacing when EAT was ≥25% of the epicardium, but no sustained reentry.

## Discussion

In this study, we showed that EATs induces electrophysiological remodeling of myocardium by decreasing electrical coupling, reducing I_K1_, and depolarizing MDP. This results in conduction slowing and increased conduction heterogeneity. By applying those changes to an *in silico* model of human LA, reentrant arrhythmias can be induced depending on the amount of EAT.

We observed that EATs, but not SATs, slowed CV and increased conduction heterogeneity in NRVMs, without altering cell viability. This differential electrophysiological effect may be due to different molecular compositions.^10^ These data add to the observation that pericardial AT-derived secretome moderately slows conduction in HL-1 cells.^7^ However, the HL-1 cell model has unphysiologically slow conduction. Our study is the first to show that human atrial EATs slows conduction in a physiologically relevant model of cultured myocytes.

AF induces structural and electrical remodeling of the atria.^11^ Therefore, the observed conduction changes induced by EATs derived from AF patients could be caused by a systemic response to AF and by a proinflammatory EATs. However, our observation that the EATs from non-AF donors led to similar conduction changes as EATs from AF patients argues against this idea.

We speculate that the amount of EAT on the heart determines arrhythmic vulnerability through the volume of released adipokines to the myocardium. The pathophysiological mechanisms leading to EAT accumulation are unknown. In atrial cardiomyopathy, it is hypothesized that stress signals from the failing myocardium lead to reactivation of the epicardium where epicardial cells differentiate, migrate, and give rise to adipocytes, participating in EAT accumulation. Accordingly, EAT accumulation may be secondary to paroxysmal AF in which increased EAT volume and adipokines secretion lead to remodeling and facilitate the progression of the disease toward persistent AF in a positive feedback interaction, in turn contributing to EAT accumulation. This is supported by observational studies showing that EAT volume is greater in AF than in non-AF patients^12^ and in patients with persistent AF than in patients with paroxysmal AF.^3^

We also showed that the CV decrease observed with EATs is concomitant with changes at the AP level: MDP depolarization, reduced APA, and decreased V_max_. The latter 2 changes are consistent with the depolarization of MDP leading to reduced availability of sodium channels, resulting in reduced sodium current and slowing of conduction. We also showed that the MDP depolarization of cardiomyocytes exposed to EATs is associated with a reduction of I_K1_. Acute incubation (2–4 hours) of cardiomyocytes with isolated rabbit epicardial adipocytes also leads to a reduction of I_K1_ and a less negative RMP.^6^ Our study confirms and adds to these observations by demonstrating that the reduction in I_K1_ and the depolarization of MDP after EATs are accompanied by decreased expression of *Kcnj2*, the gene encoding Kir2.1 channels and mediating I_K1_. This suggests that components in EATs negatively regulate *Kcnj2* transcription, resulting in reduced Kir2.1 channels, decreased I_K1_, and therefore depolarized cardiomyocytes. The slight prolongation observed in APD_90_ is consistent with a decrease of I_K1_.

Lateralization of connexin40 has been described in myocardial regions with large amounts of EAT.^7^ Because electrical coupling is essential for conduction, we assessed the expression of the gap junction protein connexin43. We show that EATs decreases the mRNA and protein expression of connexin43 by 30% and 50%, respectively, whereas connexin45 expression was unchanged. Decreased Cx43 expression can alter intercellular coupling and participate at least partially to the 25% CV reduction observed after EATs incubation.

When Cx43 expression decreases and electrical coupling is reduced, less inward current propagates to the neighboring cardiomyocytes, which increases the availability of inward current for local depolarization. As a result of this increased availability of depolarizing current, V_max_ is increased.^13^ Because EATs decreases the expression of Cx43 (Figure 3), we surmise that the observed reduction of V_max_ ( ± ) could be an underestimation of the actual effect of EATs on the sodium channel. Altered electrical coupling after EATs incubation could increase V_max_, thus masking part of the effect of the depolarization.

Finally, we demonstrated with computer simulations that the electrophysiological effects of EATs induce sustained arrhythmias when EAT covers up to 75% of the epicardium of the LA, but not at 100%. This finding emphasizes the importance of heterogeneity for the initiation of reentry. Indeed, by partially covering the atria, EAT induces electrical heterogeneity, which facilitates reentry. Finally, reducing the EAT surface from 75% to 50% of the atria or less could help to terminate reentrant arrhythmias. With this computational study, we connect the observed molecular changes driven by EATs with arrhythmia susceptibility.

The involvement of EAT in arrhythmogenesis is not restricted to atrial arrhythmias, as EAT also is present on the interventricular groove, the right ventricular free wall, and the apex.^5^ EAT volume is associated with QRS fragmentation and prolongation,^5^ suggesting its involvement in heterogeneous ventricular conduction slowing. This is supported by patient-specific simulation studies by Sung et al,^14^ who identified ablation targets in patients with postinfarct ventricular tachycardia, based on the distribution of infiltrating AT in the myocardium. Identification of patients at risk for developing arrhythmias may be based on EAT quantification.

### Study limitations

EAT and SAT biopsies were obtained from patients presenting with multiple age-dependent comorbidities such as over-weight, hypertension, and diabetes. We cannot exclude that factors associated with those comorbidities can influence the observed ionic and gap junction remodeling. However, the EATs obtained from 3 donor hearts showed the same effects on conduction as the one from AF patients, showing that several comorbidities presented by the AF patients (vascular diseases, hypertension, or heart failure) are not playing a role in the observed ionic remodeling.

We used NRVMs instead of freshly isolated adult human myocytes to study the long-term effects of EATs. Indeed, this study required the use of cultured cardiac monolayers and single cardiomyocytes that could be subjected for up to 3 days to secretome in the absence of “rundown” ionic remodeling and with an MDP as closely as possible to physiological values. NRVMs have the capacity to proliferate in the first few hours upon isolation and form monolayers, enabling accurate CV measurement, which is not the case of iPSC-CMs. In addition, freshly isolated human myocytes are frequently depolarized upon isolation and culture. iPSC-CMs display extremely low levels of I_K1_ and are also depolarized with a unphysiologically slow CV. Because I_K1_ plays an important role in EAT-induced arrhythmogenicity, this precludes the use of these cell models. Instead, confluent NRVMs monolayers exhibit high electrical cell-to-cell coupling and good maturation. Finally, CV is about 20 cm/s in NRVMs, which is twice that of iPSC-CMs and is closer to transverse CV in human atrial tissue. Despite ion current differences between ventricular and atrial cardiomyocytes, the APD of NRVMs closely resembles that of human atrial cardiomyocytes.

## Conclusion

EATs decreases intercellular electrical coupling, depolarizes the resting membrane of cardiomyocytes, and slows conduction, thus facilitating reentrant arrhythmias.

## Supplementary Material

Video 1

Supplementary Data
