# Supplementary material for: Secretome of atrial epicardial adipose tissue facilitates reentrant arrhythmias by myocardial remodeling
Source: Heart Rhythm. Author manuscript; Available in PMC 2023 Sep 1. (PMC9558493; doi:10.1016/j.hrthm.2022.05.011)
Supplement: Supplementary Data [file NIHMS1836820-supplement-Supplementary_Data.docx]

**SUPPLEMENTAL MATERIAL**

**Supplemental Methods**

**Human adipose tissue collection**

Thirty patients with symptomatic, persistent or long-standing persistent AF undergoing video-assisted thoracoscopic pulmonary vein isolation (VATS-PVI) were recruited at the Amsterdam UMC, University of Amsterdam, between 2019 and 2022. Written informed consent was obtained from all participants^1^. The study was conducted in accordance with the Declaration of Helsinki as revised in 2013 and with the Medical Research Involving Human Subjects Act (WMO) and other local laws and regulations.

AF was documented on ECG, Holter or pacemaker electrogram at least once in the 12 months preceding surgery. Patients with left ventricular ejection fraction <30% and severe heart failure were excluded from the study. The clinical characteristics of patients are listed in Supplemental Table 1.

During VATS-PVI the left atrial appendage (LAA) was resected as standard procedure. The atrial tissue was collected and the epicardial adipose tissue overlying the LAA was removed. A biopsy of approximately 1cm^3^ of subcutaneous adipose tissue (SAT) was obtained from one of the thoracoscopic entry sites. Adipose tissue was transported in phosphate-buffered saline to the laboratory.

**Donor hearts**

All studies using human hearts were approved by the Institutional Review Board (Office of Human Research) at the George Washington University. Three de-identified donor human hearts were received from Washington Regional Transplant Community (WRTC) in Falls Church, Virginia, USA. All hearts were rejected for transplantation and donated to research. Hearts were arrested by perfusion with ice-cold cardioplegic solution in the operating room and transported to the laboratory for dissection and subsequent EAT secretome extraction. The available clinical information of the donors are shown in the Supplemental Table 1.

**Adipose tissue secretome collection**

Adipose tissue was rinsed in phosphate-buffered saline, dissected to remove any blood or connective tissue, and minced into 1-2mm^3^ explants. Each explant piece was transferred to a well of a 96-well plate and incubated in 100 μL of culture medium for 24H at 37°C and 5% CO_2_ in order to stimulate adipokine and secretome release^2^ (Figure 1A). Adipose tissue secretome was then collected, pooled, filtered (0.45 µm) and stored at -80°C.

Before their use on NRVMs, the SAT and EAT secretome samples were normalized in order to obtain the same amount of EAT/SAT tissue per secretome sample, which was set to 10 mg of adipose tissue per mL of secretome. The dilutions of the EAT and SAT secretome samples were similar: dilution factor of 2.2 for EAT secretome and 2.9 for SAT secretome.

**Neonatal rat ventricular myocytes isolation**

All animal experiments were approved by the local Animal Experiments Committee (Academic Medical Center, University of Amsterdam) and carried out in compliance with the Guide for the Care and Use of Laboratory Animals and in accordance with national and institutional guidelines. Neonatal rat ventricular myocytes (NRVMs) were isolated from 1-to-2-day-old Wistar rats (Janvier labs) as previously described^3^. Pups were anesthetized by isoflurane and hearts were excised after decapitation. Ventricles were cut into pieces and dissociated with trypsin (1 mg/mL;Sigma), and collagenase type 2 (1 mg/mL;Worthington). Cells were collected and resuspended in culture medium (M199 medium, Gibco) supplemented with 10% heat inactivated fetal bovine serum (FBS, Gibco), 1% HEPES (Gibco), 5000 U/L penicillin-G (Sigma), 2 mg/L vitamin B12 (Sigma-Aldrich), 3.5 g/L glucose, 1% non-essential amino acids (Gibco), 1% L-glutamine (Gibco). The cell suspension was pre-plated to minimize fibroblast contamination. The remaining myocardial cells were plated on microelectrode arrays (MEAs, Multi-Channel Systems MCS GmbH, Reutlingen, Germany) coated with fibronectin (125 µg/mL BD Biosciences, Breda, The Netherlands) at a density of 1.4x10^5^ cells per cm^2^. NRVMs were cultured at 37°C and 5% CO_2_. After 48H of culture in medium supplemented with 10% FBS, cells were cultured in DMEM (Gibco) supplemented with 2% FBS (Gibco), 1% HEPES (Gibco), 5000 U/L penicillin-G (Sigma), 2 mg/L vitamin B12 (Sigma-Aldrich), 4.5 g/L glucose, 1% non-essential amino acids (Gibco), 1% L-glutamine (Gibco).

**Cell culture with cardiac and subcutaneous adipose tissue secretome**

Cardiomyocyte conditioned medium (CCM) was collected after culturing NRVMs for 24H and used as a control for EAT or SAT secretome. CCM was filtered (0.45 µm) and stored at -80°C in the same conditions as the adipose tissue secretome.

Two days after plating, NRVMs were incubated with CCM or EAT secretome, or -in a different experiment- CCM or SAT secretome, at a ratio of 1:1 with fresh culture medium. In order to obtain enough volume, secretome was pooled as a batch from maximum 4 patients for each experiment. For each NRVM isolation, a batch of EAT or SAT secretome was used for incubation. For the electrophysiological experiments (MEAs and patch-clamp), 7 batches of EAT secretome from 21 patients in total was used and incubated on NRVMs from 7 different isolations (Supplemental Table 2). For the RNA, protein collection, apoptosis assay and immunostaining, the EAT secretome from 17 patients was incubated in 6 batches on NRVMs from 6 different isolations. A summary of the EAT and SAT secretomes samples used for each subpart of the study can be found in Supplemental Table 2. CCM, SAT and EAT secretome were refreshed daily to avoid nutrient deficiency.

After 72H of incubation, NRVMs were collected for RNA extraction, proteins, and fixed for immunostainings as detailed below. Electrophysiological experiments were performed 48H after the start of incubation for patch-clamp and 72H after the start of incubation for electrophysiological mapping. Electrophysiological measurements were performed 10 minutes after replacing the EAT and SAT secretomes by a modified Tyrode solution in order to exclude any acute effects of the secretomes.

**Immunofluorescence assays**

NRVMs were seeded at a density of 1.4x10^5^ cells per cm^2^ on fibronectin-coated glass coverslips. After incubation with EAT secretome or CCM for 72 hours, cells were fixed in 4% paraformaldehyde, washed and permeabilized (0.2% Triton X-100 in PBS), blocked with 4% goat serum and incubated overnight with the primary antibodies in a humidity chamber at 4°C. Coverslips were incubated for 1h with secondary antibodies at room temperature, followed by DAPI (Molecular probes) staining. Images were acquired with a Leica TCS SP8 X unit mounted on a Leica DMI6000 inverted microscope.

**qRT-PCR**

RNA was extracted from cultured NRVMs with TRI reagent (Sigma-Aldrich) following manufacturer’s protocol. RNA was treated with DNAse I (Invitrogen) and subsequently used to generate cDNA using Superscript II (Invitrogen). qRT-PCR was performed on a LightCycler 480 (Roche) with SYBR Green I Master (Roche). Results were analyzed using LinRegPCR software. Primer sequences are included in Supplemental Table 3. The geometric mean of the reference genes HPRT1, EEF1E1 and GAPDH was used to calculate the relative gene expression. Gene expression was normalized to the control condition in each NRVM isolation.

**Protein isolation and Simple Western analysis.**

Cells were lysed and sonicated in RIPA buffer (50mM Tris-HCl pH8, 150mM NaCl, 1% NP-40, 0.2% sodium deoxycholate, 0.1% Sodium Dodecyl Sulfate, 1 mM Sodium Orthovanadate (Na_3_VO_4_), 1 mM PMSF) supplemented with protease inhibitor cocktail (Roche). Protein concentration was measured by BCA assay (Pierce). Proteins were separated by size and detected with a WES system (ProteinSimple, San Jose, CA, USA) using a 12-230 kDa separation module with the primary and secondary antibodies mentioned in Supplemental Table 4. Protein signal analysis and quantification was performed with the Compass software v.4.0.0 (Protein Simple).

**Live cell analysis: confluence, cell death and apoptosis**

NRVMs were isolated as previously described and seeded at a density of 2,3 x 10^5^ cells/well in 24-well tissue culture-treated plates coated with fibronectin. 48H after cell isolation, culture medium was replaced by the EAT secretome or CCM containing 0.25 μg/ml recombinant Annexin V-ATTO-488 (Adipogen Life Sciences) and 300 nM YOYO-3-612/631 (Invitrogen). Annexin V probe was used to measure phosphatidylserine exposure during apoptosis. YOYO-3, a membrane-impermeable DNA dye, was used to measure permeabilized apoptotic and dead cells. Cell confluence was quantified over time by phase contrast. 72H after incubation with EAT secretome or CCM, experiments were performed using the IncuCyte ZOOM (Essen Bioscience, Ann Arbor, MI, USA) as previously described^4-6^. Using the x20 objective, sixteen planes of view were collected per well with at least 5 wells per group (EAT or CCM). Data from phase contrast, green channel (Excitation: 440/480 nm; Emission: 504/544 nm), and red channel (Ex: 565/605 nm; Em: 625/705 nm) were collected. The total fluorescent areas of Annexin V and YOYO-3 in each image were analyzed to determine the apoptotic and dead cells. Fluorescent events were processed and analyzed using the IncuCyte ZOOM software as previously described^7^. Processing definitions for Annexin-V(488) and YOYO-3(612/631) labelled cells were defined as follows: Channel: Green; Top-Hat; Radius: 100 μM, Threshold: 2.0 RCU; Edge Sensitivity: 0; Pixel Adjust: 0; Area: >9 μm. Channel: Red; Top-Hat; Radius: 100 μM, Threshold: 2.0 RCU; Edge Sensitivity: 0; Pixel Adjust: 0; Area: >11 μm.

**Electrical mapping and microelectrode measurements**

Electrical mapping was performed as previously described^3^. Briefly, 10 minutes before measurements, NRVMs culture media/secretome was replaced by a modified Tyrode’s solution (36.5°C) containing (mM): NaCl 140, KCl 5.4, CaCl2 1.8, MgCl2 1.0, glucose 5.5, HEPES 5.0; pH 7.4 (adjusted with NaOH). NRVMs cultured on multi-electrode arrays (MEAs)(60 electrodes terminals) were stimulated using a bipolar extracellular stimulus electrode (twice diastolic stimulation threshold, 1-2ms pulse). At each local electrogram, activation time (AT) was determined as the interval from the stimulation artefact to the minimum derivative of the local QRS, and used to construct activation maps. Conduction velocity was determined along lines perpendicular to isochronal lines by dividing the distance by the difference in local AT. Heterogeneity in conduction was measured based on the method described by Lammers et al^8^. Following electrical mapping, spontaneous action potentials (APs) were measured in NRVMs monolayers using glass pipette microelectrodes (Harvard apparatus GC100F- 10). These micro-electrodes were filled with 3M KCl, typical tip resistance was 15-25 MΩ. An AgCl covered silver wire was used as a reference electrode. Maximum diastolic potential was taken as the most negative membrane potential recorded and maximal action potential (AP) upstroke velocity (V_max_) was taken as maximal dV/dt. AP duration was determined at 20, 50, and 80% (APD_20_, APD_50_, APD_80_) of the AP repolarization. All signal analysis were performed using a custom-made data analysis program written in Matlab 2006b (The MathWorks Inc, Natick, MA)^9^.

**Patch clamp experiments**

**Current and voltage clamp measurements**

Coverslips seeded with single-cell NRVMs at a density of 2,5x10^4^ cells/cm^2^ were incubated 48H with EAT secretome, SAT secretome or CCM instead of 72H like the NRVMs monolayers to ensure maintenance of single-cell configurations and sufficient membrane quality to perform patch-clamp. Coverslips were put in a recording chamber on the stage of an inverted microscope (Nikon Diaphot, Melville, New York, NY, USA) and superfused with modified Tyrode’s solution (36±0.2°C) containing (mM): NaCl 140, KCl 5.4, CaCl_2_ 1.8, MgCl_2_ 1.0, glucose 5.5, HEPES 5.0; pH 7.4 (adjusted with NaOH). Action potentials and membrane currents were measured using the amphotericin-perforated patch-clamp technique with an Axopatch 200B amplifier (Molecular Devices, Sunnyvale, CA, USA). Signals were low-pass filtered with a cut-off frequency of 2 kHz and digitized at 5 (spontaneous APs), 40 (paced APs), and 5 (membrane currents) kHz. Voltage control, data acquisition, and analysis were accomplished using custom software. Pipettes (resistance 2–3 MΩ) were pulled from borosilicate glass capillaries (Harvard Apparatus, UK) and filled with solution containing (mM): K-gluconate 125, KCl 20, NaCl 5, amphotericin-B 0.44, HEPES 10; pH 7.2 (adjusted with KOH). Cell membrane capacitance (C_m_) was determined by dividing the time constant of the decay of the capacitive transient in response to 5 mV hyperpolarizing voltage clamp steps from −40 mV by the series resistance. C_m_ amounted to 37.9±4.9 and 44.8±5.9 pF (mean±SEM; P>0.05) for CCM (n=10) and EAT-treated (n=11) cardiomyocytes, respectively. Series resistance was compensated by ≥75% and potentials were corrected for the calculated 15 mV change in liquid junction potential^10^.

NRVMs are spontaneously active^11^. We measured spontaneously APs as well as APs elicited at an overdrive stimulus frequency of 4 Hz (with 3-ms, 1.2× threshold current pulses through the patch pipette) to exclude potential frequency dependent AP effects. We analysed the cycle length, maximal diastolic potential (MDP), maximum AP upstroke velocity (V_max_), AP amplitude (APA), and APD at 20, 50, and 90% repolarization (APD_20_, APD_50_, and APD_90_, respectively). Parameters from 10 consecutive APs were averaged. APs measurements were followed by voltage clamp experiments to determine which changes in current(s) underlay the AP differences in cardiomyocyte conditioned medium (CCM) and epicardial adipose tissue (EAT) secretome-treated cardiomyocytes. To make a direct comparison between MDP and background K^+^ currents within one cell, and to ensure that AP shapes and the remaining viable cardiomyocytes in the recording chamber stay undistorted for biophysical analysis, these measurements were performed

without specific channel blockers or modified solutions. Membrane currents were examined by 500 ms voltage-clamp steps every 2s to membrane potentials ranging from -120 to +40 mV from a holding potential of -40 mV (Figure 2D, inset). The inward rectifier K^+^ current (I_K1_) and delayed rectifier K^+^ current (I_K_) were defined as the quasi steady-state current at the end of the voltage-clamp steps at potentials negative or positive to -30 mV, respectively. The L-type Ca^2+^ current (I_Ca,L_) was defined as the difference between the peak inward current and the current amplitude at the end of the 500 ms depolarizing voltage clamp step. All currents were normalized for C_m_.

**Modeling study**

***Human LA model***

An image-based computational model of the human left atria (LA), as described before^12-14^, was used to elucidate the effect of EAT secretome on arrhythmia vulnerability. Tissue conductivities were assigned to the LA model according to previous studies, see Table 2 in Bayer et al.^12^ to produce a longitudinal and transverse conduction velocity of 97 cm/s and 45 cm/s, respectively. To study the isolated effects of EAT secretome on arrhythmia vulnerability, fibrosis was not included in the LA model, nor further reductions in tissue conductivity representing a reduction in connexin 43 throughout the LA in AF patients.

***EAT regions and remodeling***

EAT regions were defined on the epicardial layer of the LA model to match EAT regions identified in imaging studies^15, 16^. Using CARPentry Studio by NumeriCor GmbH (www.numericor.at), EAT regions were interactively labeled by manually selecting mesh elements in the LA model. To investigate how the quantity of EAT affects arrhythmogenesis, mesh elements were either added or removed along the boundary of all EAT regions until the EAT percentage of the epicardium was 25, 50, 75 or 100%.

Based on the electrophysiology study results, within EAT regions the maximum conductance of the inward rectifier K^+^ current (I_K1_) was reduced by 35% and intracellular K^+^ concentration ([K]_i_) reduced by 50% in order to obtain the experimentally observed lower (less negative) resting membrane potential. Within EAT regions, longitudinal and transverse tissues conductivities were reduced until CV decreased by 25% from baseline in all directions (g_l_=0.4 S/m produces a longitudinal CV of 97 cm/s, and g_t_=0.107 S/m produces a transverse CV of 45 cm/s), as observed in the NRVMs monolayers (Figure 1). These reductions in CV are assumed to be the consequence of altered electrical coupling from reduced Cx43 expression observed in the experiments. Because EAT was obtained from patients with persistent AF, persistent AF remodeling with shortened AP duration (APD) was included on top of these changes, according to Table 2 in the online supplement of Bayer et al.^12^

Arrhythmia vulnerability was studied by applying an arrhythmia induction protocol derived from clinical studies^17^. Sinus rhythm was simulated by pacing (at the first site of SR-activation) with a cycle length of 700 ms until steady-state was reached. Then, the top of the right superior pulmonary vein was burst paced with a cycle length beginning at 150 ms, then increased by 5 ms until either reentry or a 1:1 stimulus to capture occurred. The coupling interval of the burst pacing following baseline SR pacing was set to the minimum coupling interval with capture.

***Simulation Platform***

The LA monodomain simulations were performed using the openCARP cardiac electrophysiology simulator (opencarp.org^18^) in parallel on two Intel Xeon E5-2680 V4 CPUs each @2.4 GHz with 14 cores and 1.5 TB of memory. All simulations used a time step of 20 μs and a temporal output of 5 ms.

**Supplemental Tables**

**Supplemental Table 1. Patients and donors characteristics**

| *Clinical characteristics* | *AF- patients* | | *Donor hearts* | |
| --- | --- | --- | --- | --- |
|  | *Mean±SD (range)* | *Percentage* | *Mean±SD* | *Percentage* |
|  |  |  |  |  |
| **Total** | 30 |  | 3 |  |
| **Sex** |  |  |  |  |
| Male | 19 | 63% | 3 | 100% |
| Female | 11 | 37% | 0 | 0% |
| **Age** (years) | 64±8 (39-78) |  | 53±7 (46, 52, 60) |  |
| **Body Mass index** (kg/m^2^) | 29±4 (21-38) |  | 30±5 (25, 29, 36) |  |
|  |  |  |  |  |
| **Atrial Fibrillation** |  |  |  |  |
| Persistent AF | 29 | 97% | 0 | 0% |
| Long-standing persistent AF | 1 | 3% | 0 | 0% |
| Non-AF | 0 |  | 3 | 100% |
| **Comorbidities** |  |  |  |  |
| Hypertension | 14 | 47% | 0 | 0% |
| Vascular diseases | 1 | 3% | 0 | 0% |
| Diabetes  Previous stroke  Heart failure  **Age groups**  Age>75  Age 65-74  Age <65 | 1  3  3  2  12  16 | 3%  10%  10%  7%  40%  53% | 2  1  0  0  0  3 | 67%  33%  0%  0%  0%  100% |

AF, Atrial Fibrillation.

**Supplemental Table 2. EAT and SAT secretome samples.**

|  | **EAT secretome (AF patients)** | | **EAT secretome (donors)** |
| --- | --- | --- | --- |
| **Experiment** | Electrophysiological study (MEAs and patch-clamp) | RNA and protein collection, immunostaining, apoptosis assay | Electrophysiological study (MEAs) |
| Secretome samples (#Patients) | 21 | 17 | 3 |
| Secretome concentration (mg of adipose tissue per mL) | 10 | 10 | 10 |
| Secretome batches | 7 | 6 | 3 |
| Number of NRVMs isolations | 7 | 6 | 2 |
|  |  |  |  |
|  | **SAT secretome** | |  |
| **Experiment** | Electrophysiological study (MEAs and patch-clamp) | RNA and protein collection |  |
| Secretome samples (#Patients) | 20 | 10 |  |
| Secretome concentration (mg of adipose tissue per mL) | 10 | 10 |  |
| Secretome batches | 9 | 4 |  |
| Number of NRVMs isolations | 9 | 4 |  |

**Supplemental Table 3. Primer sequences**

| **Primer** | **Sequence** |
| --- | --- |
| ATP1A1 Fw | CGGATGACAGTGGCTCACAT |
| ATP1A1 Rv | CGCTACTGCACGCTTAAGGA |
| ATP1A2 Fw | CATCATTTGCAAGACCCGGC |
| ATP1A2 Rv | AACCACCACGTGACCTTGAG |
| CACNA1C Fw | TGGGATCATGGCTTATGGCGGC |
| CACNA1C Rv | ATCAGCCAGGTTGTCCACCG |
| CACNA1G Fw | AGGCAGAGGAAATCGGCAAA |
| CACNA1G Rv | CTGTCCCCATCACCATCCAC |
| EEF1E1 Fw | TCCAGTAAAGAAGACACCCAGA |
| EEF1E1 Rv | GACAAAACCAGCGAGACACA |
| GAPDH Fw | GGTGGACCTCATGGCCTACA |
| GAPDH Rv | CTCTCTTGCTCTCAGTATCCTTGCT |
| GJA1 Fw | ACTTCAGCCTCCAAGGAGTTC |
| GJA1 Rv  GJC1 Fw  GJC1 Rv | GGTGGAGTAGGCTTGGACCT  ACTGCAGAGCAGAGCCTAGA  TGGTTTGCCCTGTTAACCAGAA |
| HPRT1 Fw | TGACTATAATGAGCACTTCAGGGATTT |
| HPRT1 Rv | CGCTGTCTTTTAGGCTTTGTACTTG |
| KCNJ2 Fw | TGTGTTACAGACGAGTGCCC |
| KCNJ2 Rv | CAGAGTTTGCCGTCCCTCAT |
| KCNJ11 Fw | ATCAGTCCAGAGGTTGGTGC |
| KCNJ11 Rv | TAATGCCCTTTCGGGACAGC |
| KCNQ1 Fw | GATCAGTCCATCGGGAAGCC |
| KCNQ1 Rv | GGTCCAGTTGTGTCACCTTGT |
| SLC9A1 Fw | AACGGCTGCGGTCCTATAAC |
| SLC9A1 Rv | CGAGACATGGTGGGTGAGTC |
| SCN1B Fw | AACACCAGCGTCGTCAAGAA |
| SCN1B Rv | TTCCGAGGCATTCTCTTGTGC |
| SCN2B Fw | CCTTGGTCCCTCAATCACCC |
| SCN2B Rv | ACTGTGACTTCCATGCTCCG |
| SCN3B Fw | CTTCCTCACCTTGTGGCTGT |
| SCN3B Rv | GCACTCAGATCACCTCAAGTCA |
| SCN5A Fw | TCTTCCGGTTCAGTGCCACC |
| SCN5A Rv | GGATGGTGCACATGATGAGCATG |
|  |  |
|  |  |
|  |  |
|  |  |
|  |  |
|  |  |
|  |  |
|  |  |
|  |  |
|  |  |

**Supplemental Table 4. List of antibodies**

| Antibody | Use | Dilution | Reference |
| --- | --- | --- | --- |
| Anti-Connexin-43 | Simple WES, IF | 1:1000, 1:250 | Sigma-Aldrich C6219 |
| Anti-Calnexin | Simple WES | 1:250 | Sigma-Aldrich 208880 |
| Anti-α-actinin | IF | 1:1000 | Sigma-Aldrich A7811 |
| Goat anti-rabbit, Alexa Fluor 488 | IF | 1:250 | Invitrogen, A-11008 |
| Goat anti-mouse, Alexa Fluor 647 | IF | 1:250 | Invitrogen, A-21235 |

IF, immunofluorescence.

**Supplemental Figures and Figure Legends**


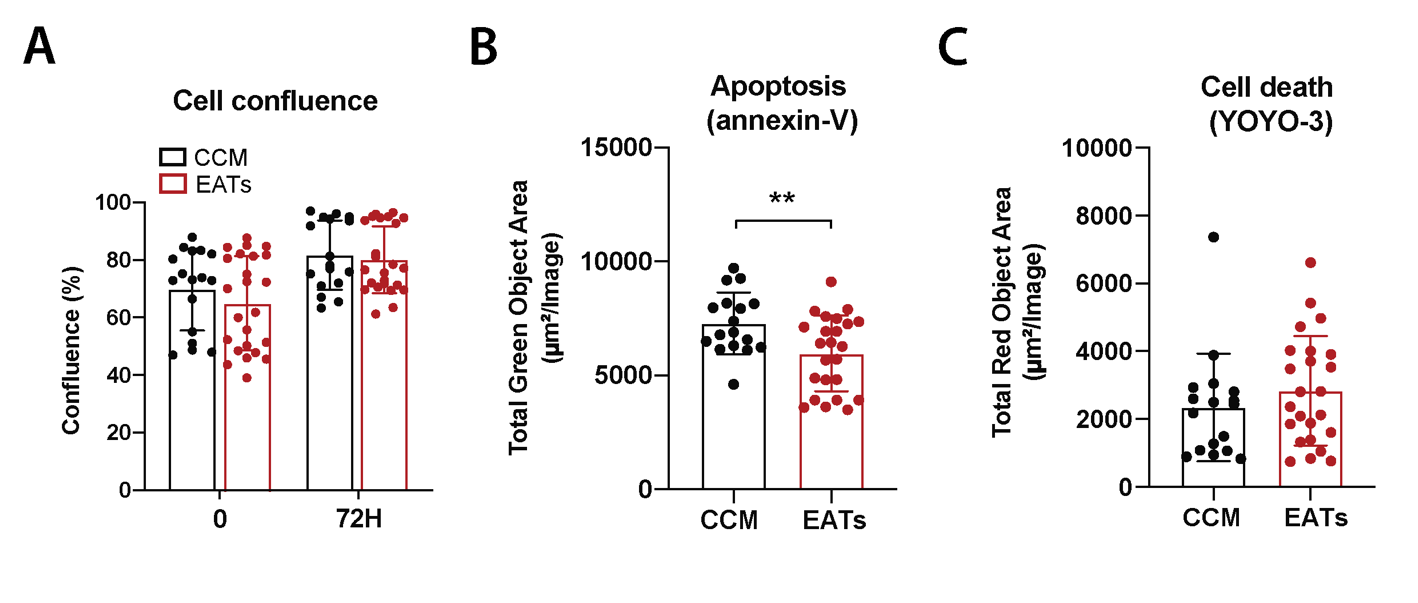


**Supplemental Figure 1. EAT secretome does not alter cell viability.**

**
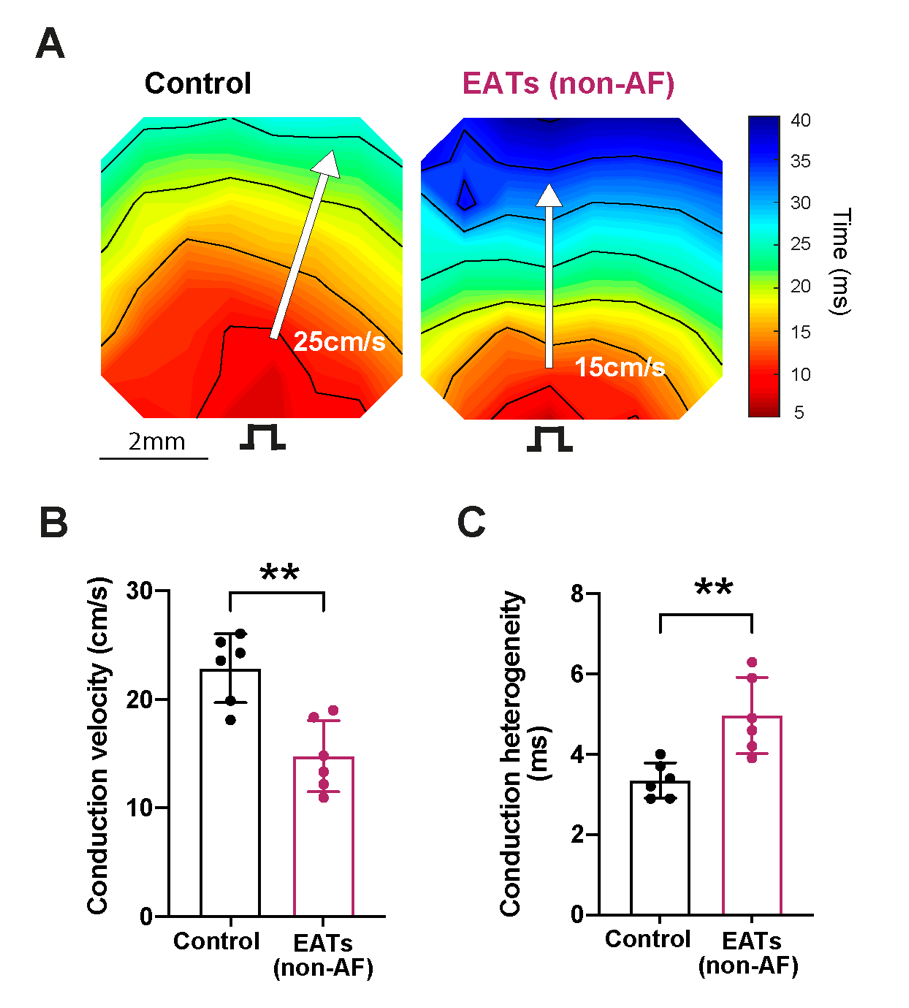
**

**Supplemental Figure 2. EAT secretome from non-AF individuals slows conduction velocity and increases conduction heterogeneity**

**(A)** Representative activation maps of NRVMs monolayers incubated 72 H with EAT secretome of non-AF individuals or control medium. Abbreviation and annotation as in Figure 1. Isochrones, 5ms.

**(B and C)** Conduction velocity and heterogeneity from NRVMs monolayers incubated 72 H with EAT secretome of non-AF individuals or with control medium. Data are mean ± SD, n=6 from two independent experiments, Student's t test.


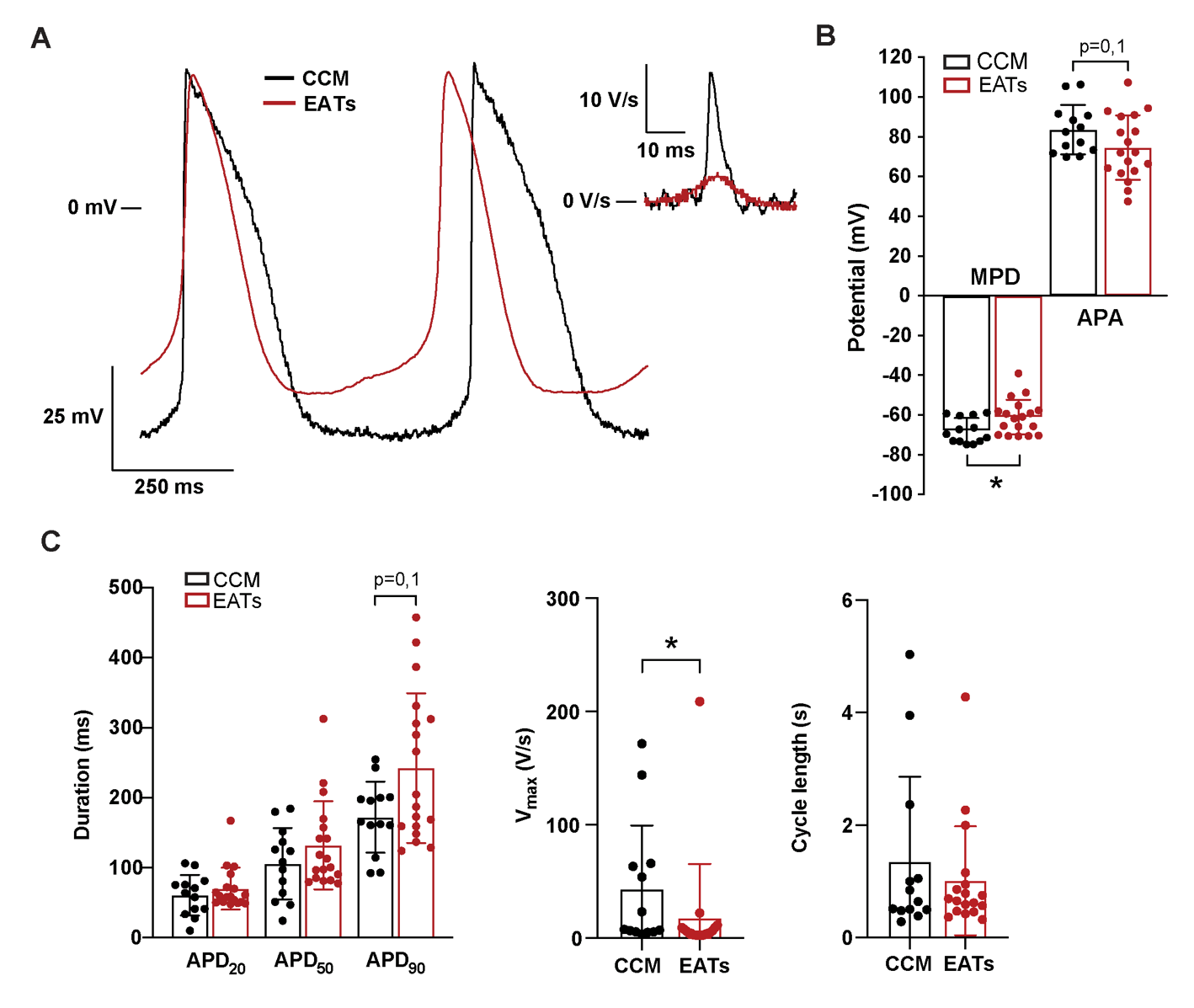


**Supplemental Figure 3. Spontaneous action potential characteristics**

**(A**) Representative spontaneous action potential recorded in single-cell cardiomyocytes after 72 H incubation with CCM or EAT secretome. Inset shows the V_max_ of action potentials.

(**B)** Spontaneous action potentials averaged values for maximum diastolic potential (MDP) and action potential amplitude (APA), *p < 0.05, nonparametric Mann-Whitney test for MDP and Student's t test for APA .

(**C**) Action potential duration in spontaneously active cells analyzed at 20, 50, and 90% of repolarization (APD_20_, APD_50_, and APD_90_), maximal upstroke velocity (V_max_) and cycle length. Data are mean ± SD from four independent experiments, *p < 0.05, nonparametric Mann-Whitney test.

EAT, epicardial adipose tissue; CCM, cardiomyocyte conditioned medium.

**
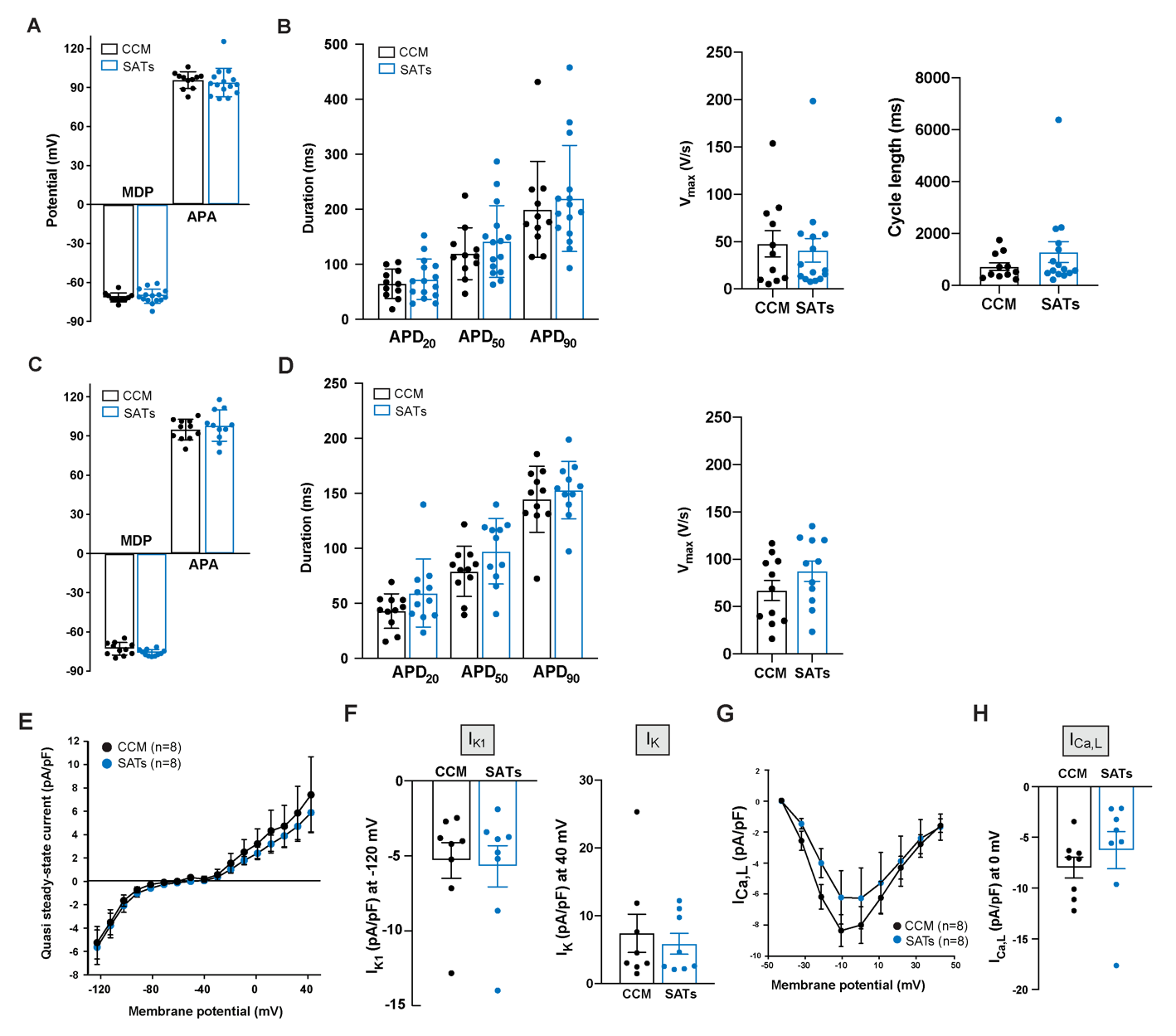
**

**Supplemental Figure 4. Patch-clamp and voltage-clamp on SAT incubates NRVMs**

(**A)** Average characteristics of spontaneous action potentials: maximal diastolic potential (MDP) and action potential amplitude (APA), **(B)** action potential duration at 20, 50, and 90% of repolarization (APD_20_, APD_50_, and APD_90_), data are mean±SD. Maximal upstroke velocity (V_max_) and cycle length, data are mean±SEM, n≥11 cells from four independent NRVMs isolation, Student’s t test for MDP, APD_20_, APD_50_; nonparametric Mann-Whitney for APA,V_max_, APD_90_, cycle length.

(**C)** Average characteristics of action potentials elicited at 4 Hz: maximal diastolic potential (MDP) and action potential amplitude (APA), **(D)** Action potential duration at 20, 50, and 90% of repolarization (APD_20_, APD_50_, and APD_90_), data are mean±SD. Maximal upstroke velocity (V_max_), data are mean±SEM. n≥11 cells from four independent NRVMs isolation, Student’s t test for MDP, APA, V_max_, APD_50_, APD_90_; nonparametric Mann-Whitney for APD_20_.

(**E)** Average current-voltage (I-V) relationships of the quasi steady-state current measured at the end of the voltage clamp steps. Data are mean±SEM.

**(F)** Current density of the quasi steady-state current measured at -120 mV (defined as I_K1_) and at 40mV (defined as I_K_). Data are mean±SEM, n≥8 cells from four independent NRVMs isolation, Student's t test.

(**G)** Average I-V relationship of L-type calcium current (I_Ca,L_). Data are mean ± SEM.

(**H)** Average current density of the quasi steady-state current measured at 0 mV, defined as I_Ca,L_. Data are mean±SEM, n≥10 cells from four independent NRVMs isolation.

SAT, subcutaneous adipose tissue; CCM, cardiomyocyte conditioned medium; MDP, maximal diastolic potential.


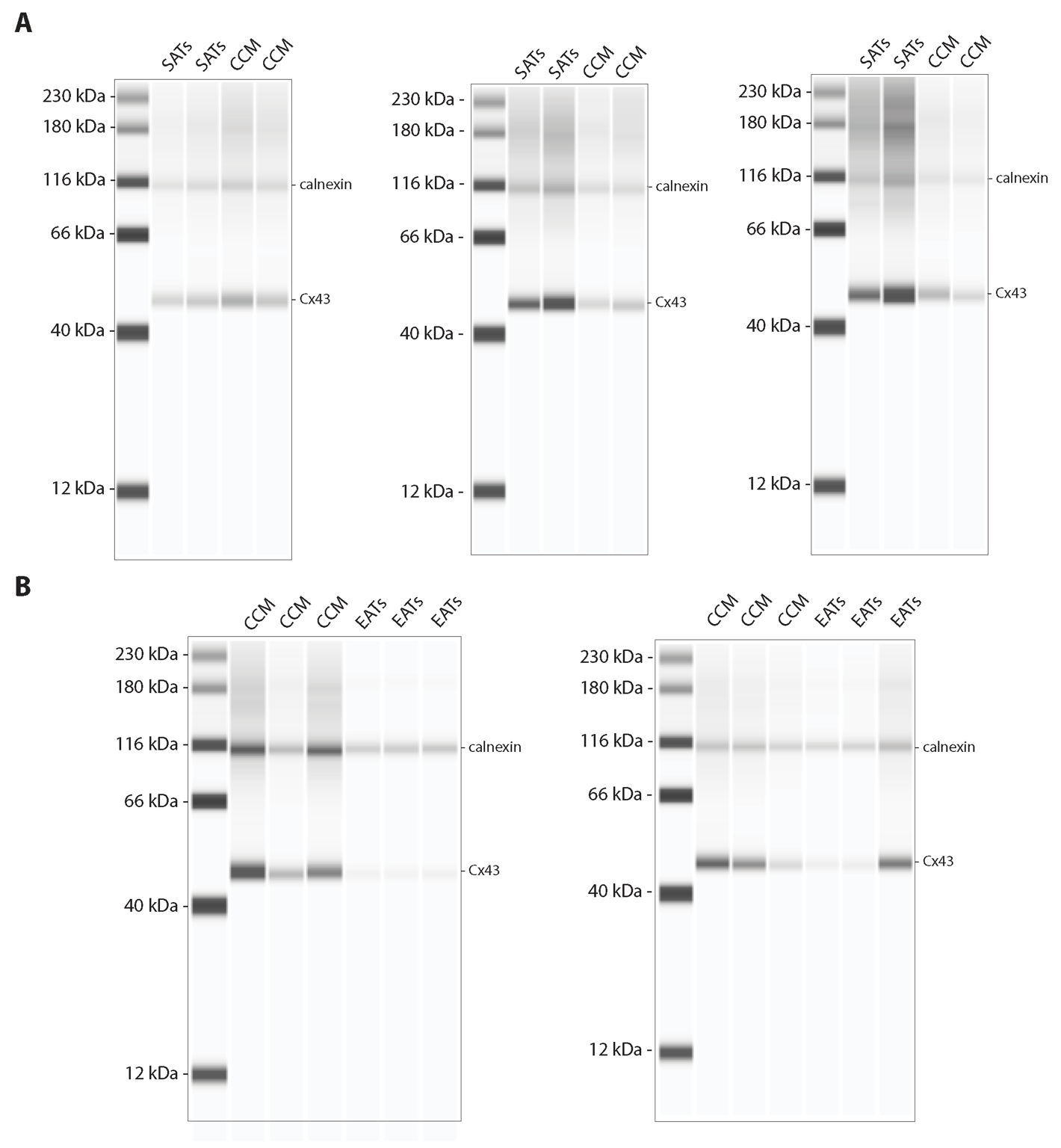


**Supplemental Figure 5. Full unedited Simple Wes blot for Figure 3G.**

(A-B) Full Simple-WES blot of Connexin 43 protein quantification in NRVMs protein lysates harvested after 72H incubation with EAT secretome from AF individuals or CCM.

SAT: subcutaneous adipose tissue, CCM: cardiomyocyte conditioned medium, EAT: epicardial adipose tissue.


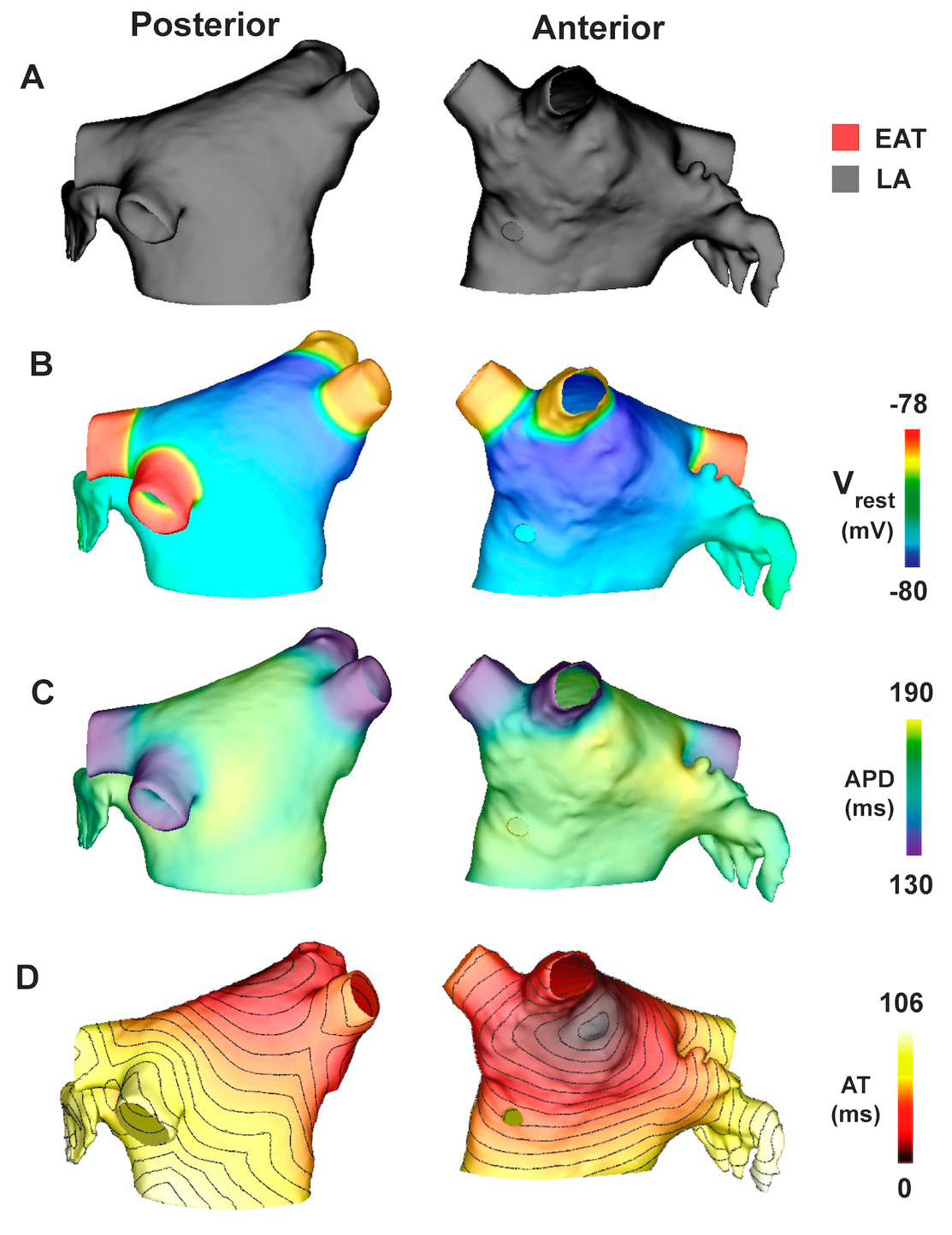


**Supplemental Figure 6. Computational Left Atrial model**

Posterior and anterior views of the computational LA model with **(A)** 0% EAT during baseline pacing with CL=700 ms from the sinus rhythm pacing site. Shown for the last beat of pacing is **(B)** resting membrane potential (V_rest_), **(C)** action potential duration (APD) at 80% repolarization and **(D)** activation time (AT) with isolines every 5 ms.

LA, Left atrium.

**Supplemental Video legend**

**Video 1.**

Videos show posterior and anterior views of the computational human left atrium model with 0, 25, 50 and 75% of EAT on the left atrium, showing membrane potential maps during burst pacing protocol.

EAT, epicardial adipose tissue; LA: left atria; V_rest_, resting membrane potential; ERP, effective refractory period; CL, cycle length.

**References**

**1.** Krul SP, Driessen AH, van Boven WJ, et al. Thoracoscopic video-assisted pulmonary vein antrum isolation, ganglionated plexus ablation, and periprocedural confirmation of ablation lesions: first results of a hybrid surgical-electrophysiological approach for atrial fibrillation. Circ Arrhythm Electrophysiol Jun 2011;4:262-270.

**2.** Viviano A, Yin X, Zampetaki A, et al. Proteomics of the epicardial fat secretome and its role in post-operative atrial fibrillation. Europace Jul 1 2018;20:1201-1208.

**3.** Smit NW, Cócera Ortega L, Végh AMD, et al. Human Cardiomyocyte Progenitor Cells in Co-culture with Rat Cardiomyocytes Form a Pro-arrhythmic Substrate: Evidence for Two Different Arrhythmogenic Mechanisms. Front Physiol 2017;8:797.

**4.** Gelles JD, Chipuk JE. Robust high-throughput kinetic analysis of apoptosis with real-time high-content live-cell imaging. Cell Death Dis Dec 1 2016;7:e2493.

**5.** Kim YE, Chen J, Langen R, Chan JR. Monitoring apoptosis and neuronal degeneration by real-time detection of phosphatidylserine externalization using a polarity-sensitive indicator of viability and apoptosis. Nat Protoc Aug 2010;5:1396-1405.

**6.** Bae SY, Guan N, Yan R, et al. Measurement and models accounting for cell death capture hidden variation in compound response. Cell Death Dis Apr 20 2020;11:255.

**7.** Elkholi R, Abraham-Enachescu I, Trotta AP, et al. MDM2 Integrates Cellular Respiration and Apoptotic Signaling through NDUFS1 and the Mitochondrial Network. Mol Cell May 2 2019;74:452-465.e457.

**8.** Lammers WJ, Schalij MJ, Kirchhof CJ, Allessie MA. Quantification of spatial inhomogeneity in conduction and initiation of reentrant atrial arrhythmias. American Journal of Physiology-Heart and Circulatory Physiology 1990;259:H1254-H1263.

**9.** Potse M, Linnenbank AC, Grimbergen CA. Software design for analysis of multichannel intracardial and body surface electrocardiograms. Comput Methods Programs Biomed Nov 2002;69:225-236.

**10.** Barry PH, Lynch JW. Liquid junction potentials and small cell effects in patch-clamp analysis. J Membr Biol Apr 1991;121:101-117.

**11.** Chan Y-C, Tse H-F, Siu C-W, Wang K, Li RA. Automaticity and conduction properties of bio-artificial pacemakers assessed in an in vitro monolayer model of neonatal rat ventricular myocytes. EP Europace 2010;12:1178-1187.

**12.** Bayer JD, Boukens BJ, Krul SPJ, et al. Acetylcholine Delays Atrial Activation to Facilitate Atrial Fibrillation. Front Physiol 2019;10:1105.

**13.** Bayer JD, Roney CH, Pashaei A, Jaïs P, Vigmond EJ. Novel Radiofrequency Ablation Strategies for Terminating Atrial Fibrillation in the Left Atrium: A Simulation Study. Front Physiol 2016;7:108.

**14.** Labarthe S, Bayer J, Coudière Y, et al. A bilayer model of human atria: mathematical background, construction, and assessment. Europace Nov 2014;16 Suppl 4:iv21-iv29.

**15.** Nakatani Y, Kumagai K, Minami K, et al. Location of epicardial adipose tissue affects the efficacy of a combined dominant frequency and complex fractionated atrial electrogram ablation of atrial fibrillation. Heart Rhythm Feb 2015;12:257-265.

**16.** Takahashi K, Okumura Y, Watanabe I, et al. Anatomical proximity between ganglionated plexi and epicardial adipose tissue in the left atrium: implication for 3D reconstructed epicardial adipose tissue-based ablation. J Interv Card Electrophysiol Nov 2016;47:203-212.

**17.** Haïssaguerre M, Jaïs P, Shah DC, et al. Spontaneous initiation of atrial fibrillation by ectopic beats originating in the pulmonary veins. N Engl J Med Sep 3 1998;339:659-666.

**18.** Vigmond EJ, Weber dos Santos R, Prassl AJ, Deo M, Plank G. Solvers for the cardiac bidomain equations. Prog Biophys Mol Biol Jan-Apr 2008;96:3-18.
